# RNA interference and validation of reference genes for gene expression analyses using qPCR in southern pine beetle, *Dendroctonus frontalis*

**DOI:** 10.1038/s41598-019-42072-6

**Published:** 2019-04-04

**Authors:** Bethany R. Kyre, Thais B. Rodrigues, Lynne K. Rieske

**Affiliations:** 1 Department of Entomology, University of Kentucky, Lexington, KY 40546-0091 USA; 2 Present Address: Greenlight BioSciences, Medford, MA 02155 USA

## Abstract

RNA interference (RNAi) is a highly specific gene-silencing mechanism that can cause rapid insect mortality when essential genes are targeted. RNAi is being developed as a tool for integrated pest management of some crop pests. Here we focus on an aggressive forest pest that kills extensive tracts of pine forests, the southern pine beetle (SPB), *Dendroctonus frontalis*. We sought to identify reference genes for quantitative real-time PCR (qPCR) and validate RNAi responses in SPB by mortality and gene silencing analysis. Using an adult beetle feeding bioassay for oral ingestion of dsRNA, we measured the expression and demonstrated knockdown of target genes as well as insect mortality after ingestion of target genes. Our study validates reference genes for expression analyses and demonstrates highly effective RNAi responses in SPB, with RNAi response to some target dsRNAs causing 100% beetle mortality after ingestion.

## Introduction

The southern pine beetle (SPB), *Dendroctonus frontalis*, is an oligophagous, tree-killing bark beetle that undergoes extreme population outbreaks that can cause devastating losses in timber^1,2^ and tourism revenue^3^, making it the most significant forest pest in the southeastern US^4,5^. SPB outbreaks, and their management, create significant disturbances, resulting in increased light availability to the forest floor, changes in temperature and soil moisture, competition, and growing space^6–9^. The influx of coarse woody debris following tree mortality affects forest succession, nutrient cycling, and wildlife associates^7^. The loss of large tracts of forest also impacts hydrologic processes that influence water quality and quantity^10^. In recent decades, changing climate patterns and lack of proactive management have allowed an unprecedented northward range expansion, and SPB is now infesting pine forests of New York, New Jersey, Rhode Island and Connecticut^11,12^. The alarming geographic range expansion, coupled with persistent outbreaks over its historic range, demonstrate the need for innovative means of managing SPB populations.

RNA interference (RNAi) technology is a novel approach to forest pest management. Introducing exogenous double stranded RNA (dsRNA) into the insect cells activates RNAi pathways that normally function to induce antiviral responses^13^. The dsRNA is bound by the DICER enzyme, cleaving it into small interfering RNAs (siRNA), which then binds to the RNA induced silencing complex (RISC), where it is digested to produce single-stranded RNA templates able to bind to complementary messenger RNA (mRNA). Binding of siRNA to mRNA induces degradation of the mRNA, preventing its translation and producing nonsensical end products. RNAi technology has proven efficacious in coleopterans, including the notable invasive forest pests, emerald ash borer (*Agrilus planipennis*)^14,15^ and Asian longhorned beetle (*Anoplophora glabripennis*)^16^. Because the process relies on matching 19 bp or more to target sequences^17^, RNAi is more target-specific than current insect suppression methods. Non-target effects can be further avoided by choosing novel target genes over highly conserved ones. Though coleopterans appear especially sensitive to RNAi^18,19^, this sensitivity is variable, and may be influenced by the beetle life stage that is targeted^20^, the process by which dsRNA is delivered^21^, and the selected target gene(s)^22^.

The analysis of target gene silencing using real-time quantitative PCR (qPCR) to evaluate relative gene expression is a standard method used to validate and confirm cellular RNAi machinery. Identifying appropriate reference gene(s) is vital for interpretation of results for other genes that are targeted in experimentation^23^. Ideal reference genes exhibit expression levels that are conserved across all cells. Reference genes (RG) are essential for proper cell metabolism (e.g. glyceraldehyde-3-phosphate dehydrogenase (GAPDH)) and/or structural integrity (e.g. actin) often exhibit stable expression making them commonly used as reference genes. The stable expression of reference genes is used as an internal control to normalize target gene expression. Though universally present in cells, reference gene expression levels can differ between organisms, and under stress and changing environments; therefore, a gene with constant non-varying expression is preferred and must be identified experimentally by screening.

We sought to validate the RNAi response in SPB by using oral delivery of dsRNA to (i) measure the expression of target genes and demonstrate gene silencing and (ii) evaluate insect mortality after dsRNA ingestion. Due to the lack of known reference genes in SPB, we screened for and identified stable genes for our gene silencing study and future gene expression studies involving SPB. Ours is the first study to validate reference genes for expression analyses and demonstrate RNAi responses in SPB.

## Results and Discussion

### Reference genes

**Figure 1:**
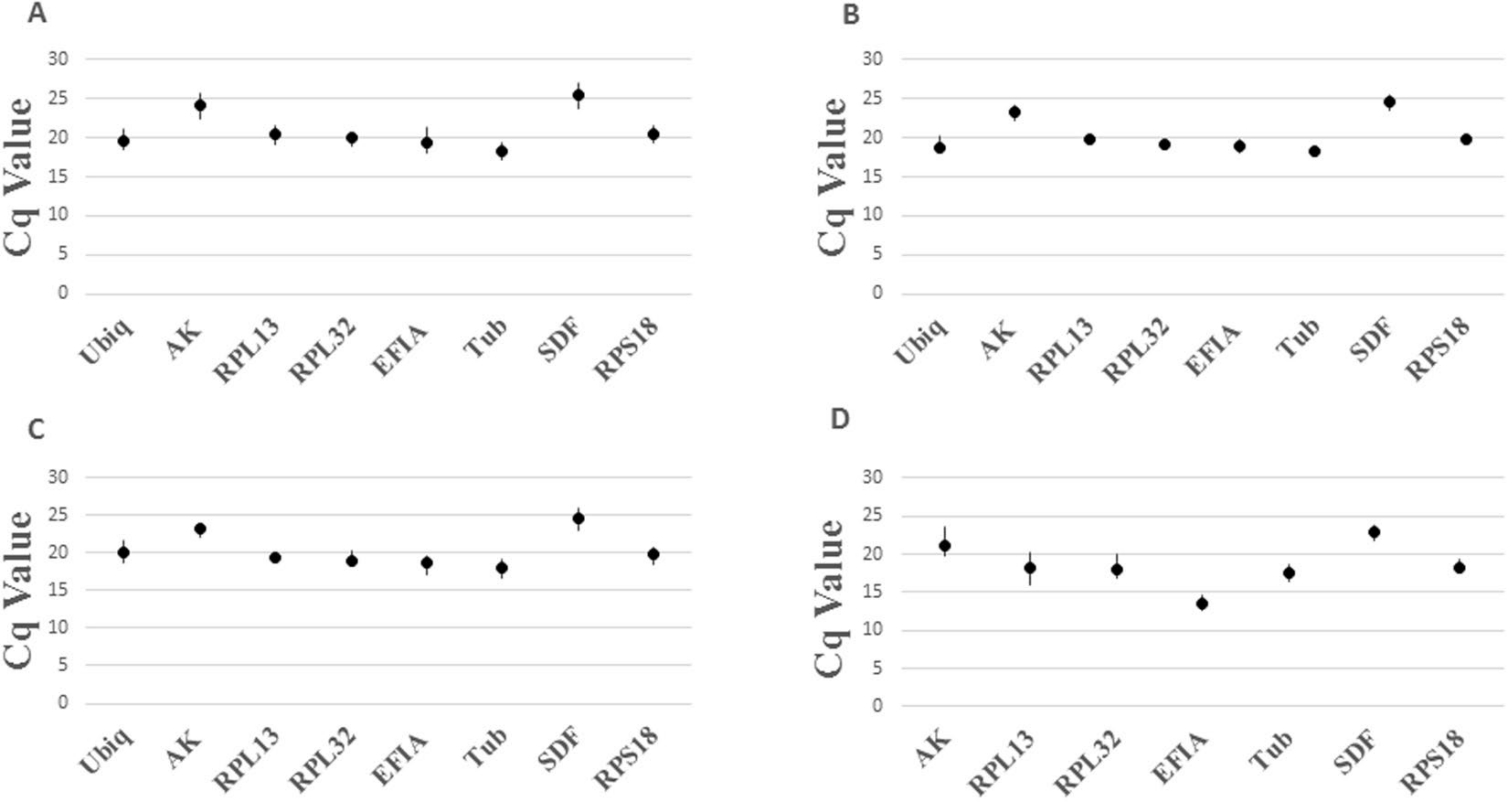
Cq values for 8 candidate reference genes in four independent experiments: (**A**) temperature, (**B**) light, (**C**) sex, (**D**) RNAi exposure. rps18 was consistently the most moderately expressed. Under RNAi exposure, sdf was the least expressed, and ef1a was the most highly expressed. Bars show the maximum and minimum Cq values.

Because qPCR is highly sensitive, normalization by internal controls is essential for accurate quantification of mRNA levels. For our study, moderately expressed genes were chosen as potential reference genes because genes with extremely high or low expression are more likely to introduce variability^16^. The Cq values for all 8 RG from all four treatments, temperature, photoperiod, sex, and introduction of dsRNA, was between 18 and 26 cycles (Fig. 1A–D). Analysis of the comprehensive values for all four treatments showed succinate dehydrogenase flavoprotein subunit A *(sdf)* was the least expressed, with average Cq values between 25 and 26, while tubulin *(tub*) was the most expressed with average Cq values ranging between 18 and 18.5. Ribosomal protein S18 *(rps18)* was the most moderately expressed RG with values ranging between 19.5 and 20.5 cycles. In this study, we focused primarily on dsRNA treatment beetles, for which *sdf* was the least expressed, with average values ranging from 22 to 23 cycles, and elongation factor – 1 alpha *(ef1a)*, which was the most highly expressed with Cq values ranging between 13 and 13.5 cycles (Fig. 1D). Ribosomal protein S18 (*rps18*) was again the most moderately expressed with Cq average values ranging between 19 and 19.5 cycles.

The Cq values from qPCR analysis were analyzed using four separate algorithms: GeNorm, NormFinder, BestKeeper, and the delta-Ct method. Genes were analyzed by all four algorithms, and comprehensively across the four algorithms, as well as analyzed separately by treatment and comprehensively across all treatments (Table 1). Again, we focused primarily on results from the dsRNA treatments. GeNorm compares all genes based on a gene expression stability value (M). Under dsRNA treatment, 7 of the 8 candidate genes expressed comprehensive M values below 1.5, with *rps18* and *ef1a* both being ranked first. NormFinder ranks genes based on an overall stability value (SV) and a SV below 1 is considered acceptable. With the exception of ubiquitin (*ubiq*), all genes produced a SV less than 1, with arginine kinase (*ak*) being ranked first. BestKeeper ranks genes based on standard deviation (SD); genes with a SD above 1 are considered less stable. Under dsRNA treatment only 4 of the 8 genes produced a SD below one and *rps18* was ranked first. Using the delta-Ct method, where lower stability values are considered favorable, *ribosomal protein L32 (rpl32)* was ranked first. Using the geomean value to create a comprehensive ranking of all four algorithms, with lower values considered more stable, *rps18* was ranked first. Given that *rps18* was ranked first using two of the four algorithms as well as comprehensively (RefFinder), it was deemed the most stable and selected as a reference gene for our study. A second reference gene, *ef1a*, was also chosen based on its rankings under GeNorm (first) and Bestkeeper (second), as well as its comprehensive ranking of second. *rps18* and *ef1a* produced corroborating results during gene expression studies. Although our study focused on dsRNA treated beetles for reference gene selection, these results identify potential genes for other gene expression studies using light, temperature, and sex as parameters. *rps18* ranked highly under all treatments and has a comprehensive treatment ranking of 1. *rpl32* ranks highly under light treatments, while *rpl13* does well under temperature treatments, and *ak* ranks highly when comparing male and female gene expression (for treatment-specific rankings for each method, metadata summary, and raw Cq values, see Supplemental Materials).

**Table 1:** Final ranking of candidate reference genes from dsRNA-treated beetles according to values given by GeNorm, NormFinder, BestKeeper, and delta Ct and a comprehensive ranking by RefFinder.

Gene	GeNorm		NormFinder		BestKeeper		delta-CT		Comprehensive	
	M	R	SV	R	SD	R	SD	R	GM	R
*rps18*	0.028	1	0.81	7	0.65	1	0.94	5	2.43	1
*ef1a*	0.028	1	0.773	6	0.67	2	0.92	4	2.63	2
*rpl13*	0.617	6	0.336	3	1.44	7	1.08	7	5.66	7
*ak*	0.501	5	0.165	1	1.41	6	0.97	6	3.83	6
*sdf*	0.1	2	0.666	5	0.71	3	0.87	3	3.41	5
*rpl32*	0.326	4	0.206	2	1.11	5	0.84	1	2.66	3
*tub*	0.177	3	0.546	4	0.79	4	0.86	2	3.36	4
*ubiq*	1.16	7	2.777	8	3.1	8	2.79	8	8	8

M: gene expression stability; R: ranking; SV stability value; SD: standard deviation; GM: Geomean value.

### Analysis of target gene expression

**Figure 2:**
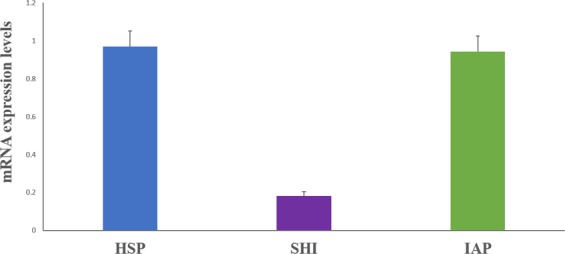
Relative expression of hsp, shi, and iap in adult SPB. Relative expression of hsp and iap is higher than that of shi (means followed by the same letter do not differ, one way ANOVA, F2,17 = 36.7, Tukey’s P < 0.0001).

Of the three target genes evaluated, heat shock protein (*hsp)*, shibire (*shi*), and inhibitor of apoptosis (*iap), hsp* and *iap* have the greatest relative expression and *shi* has the lowest (Fig. 2). There is no detectable difference in expression between *hsp* and *iap*, but both differ significantly from *shi*. Higher gene expression levels do not necessarily make *hsp* and/or *iap* better RNAi targets for inducing mortality. Highly expressed genes may produce shorter half-life proteins and thus require a higher level of expression to perform necessary functions, making them more susceptible to RNAi^24^, whereas genes with lower levels may produce longer half-life proteins, and therefore do not need to be highly expressed.

### Beetle mortality

**Figure 3:**
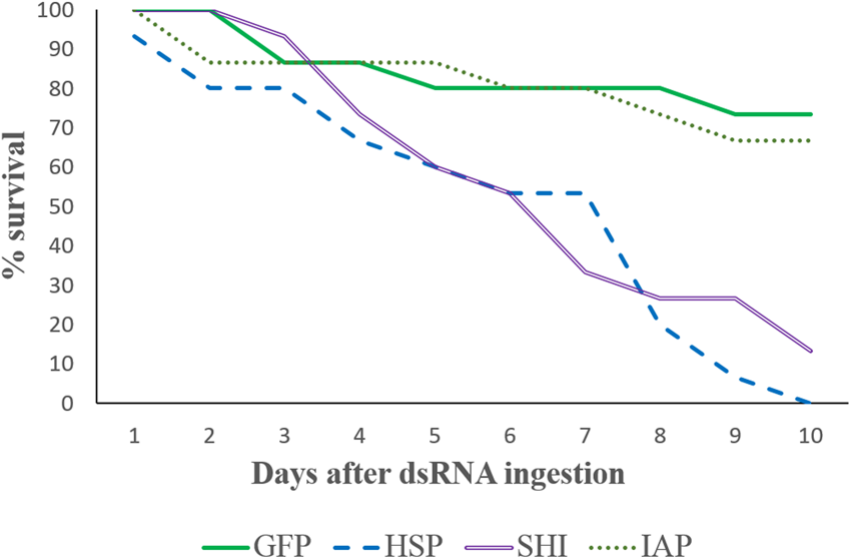
Adult SPB survival 10 days after ingesting 1 µL of 10 µL of dsRNA. At day 5, significant differences in survival were evident for beetles ingesting dsHSP and dsSHI relative to those ingesting dsIAP and dsGFP (ANOVA, F3,39 = 4.69, P = 0.0073).

With the exception of a single beetle lost in the dsHSP treatment, which may be attributed to experimental handling, no mortality was observed in any dsRNA treatment after 24 hours. After 5 days both dsHSP and dsSHI treated beetles exhibited 40% mortality, which rose to 80% and 73.33% respectively on day 8. At 10 days dsHSP treated beetles experienced 100% mortality, and dsSHI beetles experienced 86.67% mortality (Fig. 3). The difference in mortality between *hsp* and *shi* was not significant (t-test, one tailed P = 0.65), but both mortalities were significant relative to control beetles (t-test, one tailed, P = 0.0005). Mortality in dsIAP treated beetles did not differ from control beetles (t-test, one tailed P = 0.72).

### Gene silencing

**Figure 4:**
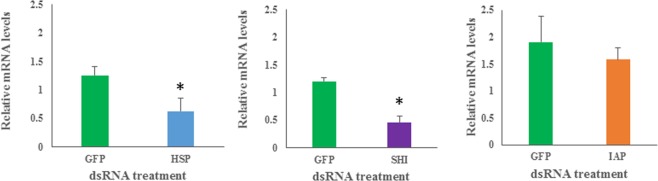
Ingestion of gene specific dsRNAs by adult SPB fed with 1 µL of 10 µg/µL of dsRNA, or dsGFP as a control, resulted in significant silencing of A) hsp (P = 0.0445) and B) shi (P = 0.0013) (one-tailed t-test), but not C) iap (P = 0.3033) (* denotes significant differences).

To assess whether beetle mortality was caused by an RNAi response, gene expression analysis was performed 24 h after dsRNA ingestion using both *rps18* and *ef1a* as internal standards, which showed corroborating results. Ingestion of dsHSP and dsSHI by adult beetles resulted in significant silencing of nearly 50% when compared to expression in control beetles (Fig. 4), whereas ingestion of dsIAP did not result in significant silencing of the *iap* gene. Our gene silencing results corroborate the mortality produced in *hsp* and *shi* treated beetles. The lack of *iap* silencing and the inability to induce mortality in *iap* treated beetles further supports that the mortality we observed was a result of gene silencing using the RNAi pathway. However, our *iap* mortality and gene silencing results differ from studies with emerald ash borer (EAB), another forest pest in which *iap* is a highly effective target gene^16^; higher concentrations, longer exposure of dsIAP, or the use of additional dsIAP fragment(s) may produce results similar to those found in EAB. The lack of significant mortality in dsIAP treated beetles corroborates results from our gene expression studies, and demonstrates that *iap* is not a suitable target gene for SPB under our experimental parameters.

In conclusion, ours is the first study to demonstrate an RNAi response in southern pine beetle, which led to 100% beetle mortality after ingestion of dsRNA. Although gene silencing in agricultural pests has been studied extensively^25–28^, an understanding of the pervasiveness and efficacy of RNAi mechanisms in forest pests is lacking. Initial work with SPB adults demonstrates that a single oral delivery of dsSHI and dsHSP, chosen based on precedence with other forest pests^16,29^ results in 86.6% and 100% mortality respectively after 10 days. These results align with studies using emerald ash borer neonates^29^ and red flour beetle (*Tribolium castaneum*)^30^. However, our study differs from that investigating EAB in that EAB neonate feeding lasted 4 consecutive days and generated 80% and 93.3% mortality. And though the *T. castaneum* study generated mortality of 100%, the beetles were injected rather than fed. Oral delivery of dsRNA is less invasive and largely reduces mechanical injury to the insect, but can be less reliable as gut morphology may prevent the delivery of the dsRNA to the midgut epithelium^31^ and dsRNA degrading enzymes can be present in the gut^32^. Our results suggest that silencing of the target genes *hsp* and *shi* is efficient in SPB, and that SPB may be particularly sensitive to RNAi. Future efforts evaluating RNAi in SPB should focus on screening additional candidate target genes to ensure selection of the most rapid and efficacious for SPB suppression. We recognize that not all effective target genes need lead to high mortality. For example, targeting genes for reproductive suppression or genes leading to a gradual decline in fitness may also prove suitable. Optimal target gene selection is essential for development of effective RNAi technologies to combat southern pine beetle, whose impacts are exacerbated by land management practices^33^, and changing temperature and precipitation regimes^34^. Dose responses and combinations of target genes should also be investigated, as these factors have played a role in RNAi efficacy in other forest pests^29^. Development and deployment of innovative gene silencing technologies such as RNAi in combating forest pests could provide a much-needed tool for natural resource managers, providing an additional component for integrated pest management while minimizing off target effects. Our study is the first step in investigating the feasibility of the use of RNAi against southern pine beetle.

## Methods and Materials

### Insects

Adult beetles were obtained from loblolly pine, *Pinus taeda*, bark samples collected from areas with high SPB populations in the southeast USA and stored in darkness at 4 °C. Infested bark was removed as needed, and placed in emergence containers consisting of a sealed, darkened 2 L container with a clear 25 ml collection tube in one end containing a moistened tissue. Emergence containers were held at 23 °C and monitored daily, and only newly emerged adult beetles were used in bioassays.

### Reference gene(s)

#### Candidate gene treatments and selection

Eight reference genes were selected as candidate reference genes: ribosomal protein S18 (*rps18*), elongation factor – 1 alpha *(ef1a*), ribosomal protein L13 (*rpl13*), arginine kinase (*ak)*, succinate dehydrogenase flavoprotein (*sdf*), ribosomal protein L32 (*rpl132*), tubulin (*tub*), and ubiquitin (*ubiq*)^16,35,36^. Gene expression was analyzed in individual adult beetles (N = 5) while controlling for four parameters: temperature, light, sex, and exposure to dsRNA, or RNAi response. Temperature treatment beetles were kept at 20 °C and 25 °C. Light treatment beetles were maintained in either total darkness or at 15:9 (L:D). Beetles maintained in both temperature and light regimes were kept in layers of bark and tissue in standard size petri dishes (60 × 15 mm) and were evaluated after three days. Male and female beetles were sexed upon emergence using the presence of pronotal grooves (female) and frontal tubercles (male)^37^ and used in experiments immediately after sexing. For RNAi exposure, individual adult beetles (N = 5) were fed four separate dsRNA treatments (*hsp, shi, iap*, and *gfp*) of 10 µg/µL in a 1% sucrose solution. Following dsRNA ingestion, beetles from each treatment were placed together in petri dishes containing damp filter paper and pine bark; dishes were oriented vertically and maintained at 23 °C with a 15:9 L:D photoperiod. Beetles were evaluated after 24 hours.

#### Stability analysis

Stability of candidate genes was evaluated by inputting the mean Cq (quantification cycle) value of each beetle per primer into BestKeeper^38^, an Excel based tool which uses pair-wise comparisons to evaluate gene stability, and into the web based tool, RefFinder^39^, which integrates four separate algorithms (Table 1) to determine the stability: GeNorm, NormFinder, BestKeeper, and the delta-Ct method. GeNorm measures stability by taking the geometric average and mean pairwise variation of all candidate genes, the results of which produce an M-score (M). Lower M-scores denote higher stability; and genes with scores greater than 1.5 are not considered^40^. GeNorm selects the best pair of genes, rather than the best single gene. NormFinder produces an overall stability value (SV) by measuring intra and intergroup variations of candidate genes. As with GeNorm, the lower the value, the more stable the gene, with 1 being the cutoff^41^. BestKeeper measures the standard deviation (SD) of each gene. Again, lower scores denote higher stability, and genes with a SD greater than 1 are considered less stable^42^. The delta-Ct method is a comparative method that estimates stability based on delta-Ct value variation. Again, lower scores denote greater stability^43^. RefFinder calculates a comprehensive final ranking of the geometric mean of the four algorithms with smaller geometric means denoting higher stability^44^.

### RNA extraction, cDNA synthesis, and qPCR

Total RNA was isolated from whole beetles with TRI Reagent RT (Molecular Research Center Inc., Cincinnati, OH), RNA integrity was verified using gel electrophoresis and absorbance was measure at 260/280 and 230/280. cDNA was synthesized using SuperScript¨ III Reverse Transcriptase (Invitrogen, Carlsbad, CA) according to manufacturer’s instructions at a concentration of 3000 ng/ml and used as a template for the qPCR standard curve, constructed using a 5-fold dilution. Each qPCR sample contained 1 µL of 3000 ng/µL synthesized cDNA (diluted 1:1), 0.2 µL of each primer (forward and backward), 3.6 µL of nuclease free ddH_2_O, and 5 µL of SYBR Green PCR Master Mix (Applied Biosystems, USA); totaling 10 µL. All reactions were performed using SYBR Green Master Mix and amplified under the following cycling conditions: beginning cycle at 95 °C, 40 cycles at 95 °C for denaturation, followed with 30 s at 65 °C for annealing and extension, and ending with generation of a melting curve consisting of a single peak to rule out non-specific product and primer dimer formations. Each sample was repeated three times and measured using the mean Cq value. For evaluating reference genes, the mean Cq value of each sample and each primer was used as input data. For gene expression analysis the 2^*−*∆∆Ct^ method^45^ was used to calculate the relative expression level of the target gene with *rps18* and *ef1a* as reference genes. For statistical analysis, we performed a one-way ANOVA with Tukey’s HSD to evaluate differences (P < 0.05). Primers for the eight selected genes (Table 2) as well as three target genes (Table 3) were designed using Primer3Plus and validated using correlation coefficients (R^2^) and amplification efficiencies (Eff). Standard curves were constructed using 5-fold serially diluted cDNA for each pair of primers. A desired R^2^ is >0.99 and acceptable amplification efficiencies fall between 90% and 110%^46^.

**Table 2:** Candidate reference genes and corresponding primer sequences.

Gene Name	Sequence 5′3′	R^2^	Eff%
*rps18 –* Ribosomal Protein S18	GCCCTCTTGTTCAAATCCAC	0.99	98.6
	CTTAACGGCCATCAAAGGAG		
*ef1a –* Elongation Factor – 1 alpha	TCCAAGAGGTGGGAATTCAG	0.99	100
	GATCGTCGTTCAGGAAAAGC		
*rpl13 –* Ribosomal Protein L13	AACCCCAAGAGGAAAGGATG	0.99	96.2
	CCAGGCGCTTTTTAGAACTG		
*ak –* Arginine Kinase	GATGGGGTCGAACAAATCAG	0.99	98.3
	AGAAAACGTCCTTCGGTTCC		
*sdf –* Succinate dehydrogenase flavoprotein subunit A	AGTTGGCAGAGACCCATTTG	0.99	100
	CGCGTGTCGAGATGTTAAAGC		
*rpl32 –* Ribosomal Protein L32	ATTGTGGACCAGCACTTTCC	0.99	99.3
	TATCGACAACAGGGTGAGGAG		
*tub -* Tubulin	TCCTGATCCTGTCCAAAACC	0.99	99.3
	TGATCACCGGAAAGGAAGAC		
*ubiq -*Ubiquitin	ATTGGAAGATGGACGCACTC	0.99	95.7
	TGCCAGTCAAACTCTTCACG		

R^2^: Correlation Coefficients; Eff: Amplification efficiency.

**Table 3:** Primer sequences and amplicon size for qPCR and dsRNA synthesis of the target genes *hsp, shi*, and *iap*.

Gene	Primer	Primer Sequence (5′-3′)	Amplicon (bp)
*hsp –* heat shock protein	F-qRNA-HSP	ACACGCACACTCGTTCTCAC	139
	R-qRNA-HSP	TACGCGTACTCGCTGAAGAA	
	F-dsRNA-HSP	**TAATACGACTCACTATAGGGG**TGCAG CAACTGGTCAAAGA	351
	R-dsRNA-HSP	**TAATACGACTCACTATAGGGG**TCTTT GGTCATGGGACGTT	
*shi –* shibire	F-qRNA-SHI	AGTTCGCCGTTGATGAAATC	86
	R-qRNA-SHI	TCGAGCAGGGCTTTATGTCT	
	F-dsRNA-SHI	**TAATACGACTCACTATAGGG**TAGATC GGTGTCAGTTCCCC	379
	R-dsRNA-SHI	**TAATACGACTCACTATAGGG**GCGAGC GCGTTTTCTATTAC	
*iap –* inhibitor of apoptosis	F-qRNA-IAP	TTTCGTTTGATGCTCGACTG	109
	R-qRNA-IAP	TCTTCGCCTGTCCTGTCTTT	
	F-dsRNA-IAP	**TAATACGACTCACTATAGGG**GTCCCGC TCATCCAGATAAA	370
	R-dsRNA-IAP	**TAATACGACTCACTATAGGG**TTTTGCCT CTTTCGCACTTT	

Bold letters denote promoter sequences of T7 RNA polymerase.

### dsRNA synthesis from cDNA

Gene specific primers (Table 3) designed using SnapDragon were used to amplify dsRNA templates, and run under normal PCR conditions as follows: 4 min at 94 °C, followed by 35 cycles of 30 sec at 94 °C, 30 sec at 60 °C, and 45 sec at 72 °C. The final step is an extension incubation which takes place at 72 °C for 10 min. PCR templates were purified using a Qiagen purification kit (Qiagen, Germantown, MD). Once purification was completed, MEGAscript RNAi Kit (Thermo Scientific, Waltham, MA) was used in dsRNA synthesis, per manufacturer’s instructions. The reaction mix was then incubated at 37 °C for 14 hours; after which it underwent 30 minutes of DNase treatment at 37 °C. dsRNA was recovered by adding 2 µL (0.1 × volume) of sodium acetate and 50 µL (2.4 × volume) of 100% EtOH to the reaction mix which was then incubated at −20 °C for 2 hours. After incubation, the mix was spun at −4 °C for 30 min (14000 rpm), then washed with 750 µL of 75% EtOH and spun at −4 °C for 15 min at 13000 rpm. Once rinsed, samples were dried at 37 °C for 25 min and re-suspended in 20 µL of nuclease free H_2_O. dsRNA quality was checked using electrophoresis and quantified with a spectrophotometer (NanoDrop Technologies, Wilmington, DE). To attain a desired concentration of 10 *µ*g/*µ*L, dsRNA was dried using vacuum speed at 30 °C for 15 minutes and resuspended in nuclease free H_2_O.

### Adult beetle feeding assays

**Figure 5:**
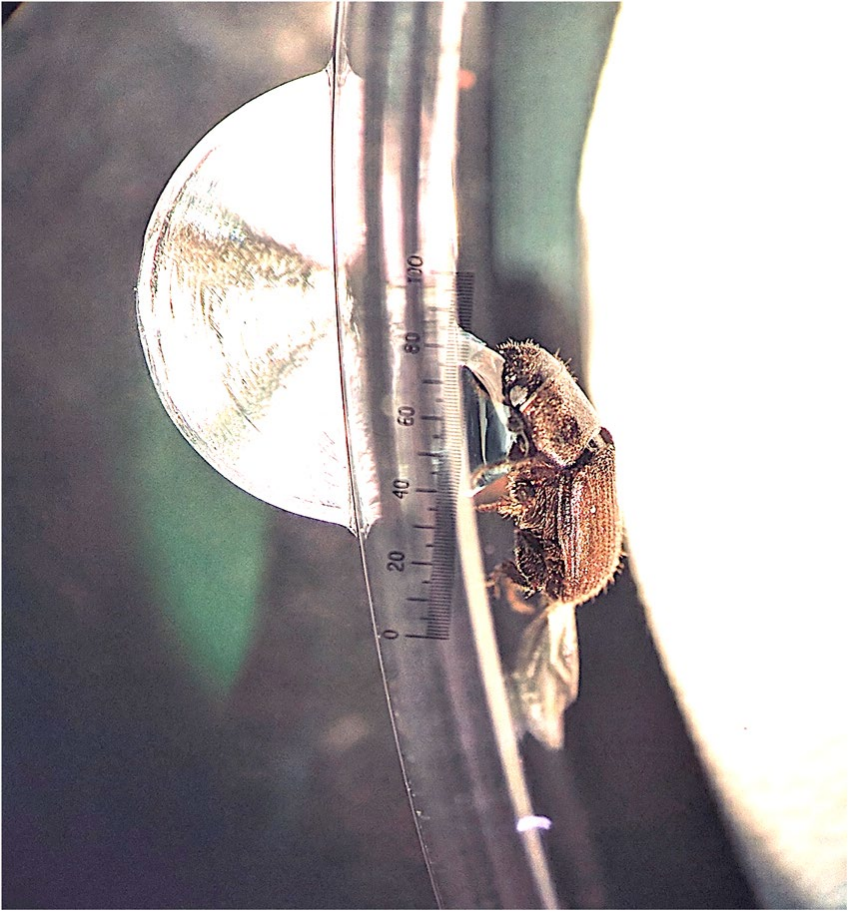
Adult SPB feeding on sucrose droplet.

To evaluate adult SPB mortality and gene silencing, adult beetles (N = 15 per treatment for mortality assay; N = 5 per treatment for gene silencing) were fed dsRNA diluted in sucrose. A 1 µL drop of 10 µg of dsRNA in a 1% sucrose solution colored with blue food coloring for easier visualization was placed on the wall of a 50 mm petri dish (Fig. 5)^15^. Individual beetles were then placed in the petri dish so that their mouthparts were in contact with the droplet and held in place with a paintbrush, applying slight pressure to the back of the head. Each beetle was held in place until the solution was consumed (3–5 min). Beetles were fed one at a time to ensure complete ingestion of the droplet.

Once dsRNA ingestion was complete, beetles from each treatment were placed together in petri dishes containing damp filter paper and pine bark; dishes were then oriented vertically beneath a clear plastic chamber and maintained at 23 °C with a 15:9 L:D photoperiod. Beetles were monitored for 10 days, with mortality being recorded every 24 hours. For gene silencing analysis, beetles were collected after the first 24 hours, at which time total RNA was extracted. For statistical analysis of beetle mortality, a one way ANOVA on non-transformed data was performed, and Tukey’s test was used to identify differences between treatments. For gene silencing analysis, data were determined to be normal (Shapiro-Wilk test, W = 0.6652), and a one-tailed t-test was used to compare differences of a single variable.

## Supplementary information

Supplemental Materials

Supplemental Data
