# Supplementary material for: RNA interference and validation of reference genes for gene expression analyses using qPCR in southern pine beetle, Dendroctonus frontalis
Source: Sci Rep. 2019 Apr 4;9:5640. doi: 10.1038/s41598-019-42072-6 (PMC6449504; doi:10.1038/s41598-019-42072-6)
Supplement: Supplementary file 1 — Supplemental Materials [file 41598_2019_42072_MOESM1_ESM.pdf]

RNA interference and validation of reference genes for gene expression analyses using RT-qPCR in southern pine beetle, *Dendroctonus frontalis*

Bethany R. Kyre[1], Thais B. Rodrigues[1,2] & Lynne K. Rieske[1*]

[*]Corresponding author (Lrieske@uky.edu)
[1]University of Kentucky Department of Entomology, S-225 Ag North, Lexington, KY 40546-0091
[2]Current address Greenlight BioSciences, Inc., Medford, MA

Supplemental Materials

Final ranking of candidate reference genes from light treated beetles according to values given by GeNorm, NormFinder, BestKeeper, and delta Ct and a comprehensive ranking by RefFinder. <u>M: gene expression stability; R: ranking; SV stability value; SD: standard deviation; GM: Geomean value.</u>

Gene	GeNorm		NormFinder		BestKeeper		delta-CT		Comprehensive	
	M	R	SV	R	SD	R	SD	R	GM	R
rps18	0.349	1	0.424	3	0.22	1	0.63	3	1.73	2
ef1a	0.522	4	0.271	2	0.54	5	0.56	2	3.16	4
rpl13	0.435	2	0.441	4	0.23	2	0.63	4	3.13	3
ak	0.619	6	0.662	8	0.76	8	0.77	7	7.48	8
sdf	0.578	5	0.489	5	0.65	7	0.67	5	5.69	6
rpl32	0.349	1	0.259	1	0.25	3	0.55	1	1.32	1
tub	0.476	3	0.514	6	0.37	4	0.68	6	4.9	5
ubiq	0.659	7	0.651	7	0.55	6	0.78	8	7.2	7

Final ranking of candidate reference genes from temperature treated beetles according to values given by GeNorm, NormFinder, BestKeeper, and delta Ct and a comprehensive ranking by RefFinder. **<u>M: gene expression stability; R: ranking; SV stability value; SD: standard deviation; GM: Geomean value.</u>**

Gene	GeNorm		NormFinder		BestKeeper		delta-CT		Comprehensive	
	M	R	SV	R	SD	R	SD	R	GM	R
rps18	0.177	1	0.256	2	0.72	1	0.43	2	1.57	1
ef1a	0.414	6	0.423	7	1.15	8	0.51	5	7.24	8
rpl13	0.27	2	0.205	1	0.78	4	0.42	1	1.86	2
ak	0.386	4	0.285	4	1.01	6	0.42	1	3.94	4
sdf	0.366	3	0.289	5	0.99	5	0.44	3	4.73	5
rpl32	0.472	7	0.6	8	0.75	3	0.65	6	6.26	7
tub	0.177	1	0.282	3	0.73	2	0.44	3	2.21	3
ubiq	0.402	5	0.355	6	1.02	7	0.47	4	6.24	6

Final ranking of candidate reference genes from male and female beetles according to values given by GeNorm, NormFinder, BestKeeper, and delta Ct and a comprehensive ranking by RefFinder. M: gene expression stability; R: ranking; SV stability value; SD: standard deviation; GM: Geomean value.

Gene	GeNorm		NormFinder		BestKeeper		delta-CT		Comprehensive	
	M	R	SV	R	SD	R	SD	R	GM	R
rps18	0.329	2	0.167	1	0.55	2	0.59	1	1.57	1
ef1a	0.356	3	0.305	2	0.79	6	0.62	4	3.72	5
rpl13	0.449	5	0.364	5	0.41	1	0.72	5	3.5	4
ak	0.317	1	0.317	3	0.67	3	0.61	2	2.06	2
sdf	0.376	4	0.607	7	0.91	8	0.73	6	6.4	6
rpl32	0.317	1	0.341	4	0.7	4	0.62	3	2.63	3
tub	0.525	6	0.524	6	0.83	7	0.81	7	6.74	7
ubiq	0.784	7	1.521	8	0.72	5	1.56	8	7.11	8

Final ranking of candidate reference genes from all treatments according to values given by GeNorm, NormFinder, BestKeeper, and delta Ct and a comprehensive ranking by RefFinder. M: gene expression stability; R: ranking; SV stability value; SD: standard deviation; GM: Geomean value.

Gene	GeNorm		NormFinder		BestKeeper		delta-Ct		Comprehensive	
	M	R	SV	R	SD	R	SD	R	GM	R
rps18	0.475	4	0.297	1	0.59	1	0.61	2	1.68	1
ef1a	0.362	2	0.314	2	0.8	5	0.6	1	2.34	2
rpl13	0.505	3	0.348	3	0.53	2	0.63	3	3.08	3
ak	0.312	1	0.43	4	0.83	6	0.64	4	3.13	4
sdf	0.312	1	0.464	6	0.92	8	0.66	5	4.12	5
rpl32	0.517	5	0.434	5	0.64	4	0.66	5	4.95	6
tub	0.556	6	0.485	7	0.63	3	0.71	6	5.66	7
ubiq	0.71	7	1.103	8	0.89	7	1.17	7	7.74	8
